# Non-Classical Aspects of Obesity Pathogenesis and Their Relative Clinical Importance for Obesity Treatment

**DOI:** 10.3390/healthcare11091310

**Published:** 2023-05-03

**Authors:** Georgia E. Samakidou, Chrysi C. Koliaki, Evangelos N. Liberopoulos, Nikolaos L. Katsilambros

**Affiliations:** Diabetes Center, First Department of Propaedeutic Internal Medicine, Medical School, National and Kapodistrian University of Athens, Laiko General Hospital, 11527 Athens, Greece; ckoliaki@yahoo.com (C.C.K.); elibero@med.uoa.gr (E.N.L.); nkatsil@med.uoa.gr (N.L.K.)

**Keywords:** obesity, ultra-processed foods, diet quality, intermittent fasting, endocrine-disrupting chemicals, inadequate sleep

## Abstract

Obesity is a chronic disease and a major public health problem due to its association with non-communicable diseases and all-cause mortality. An increased energy intake and decreased physical activity have been long recognized as the classical parameters that contribute to the development of obesity. However, several other, non-classical factors have also been associated with obesity through various complex mechanisms. Some of them are diet related, such as diet quality, dietary habits and speed of eating. Other factors are non-dietary, such as endocrine-disrupting chemicals, sleep quality and quantity, psychotropic medications and light at night. The scope of the present narrative review is to address these non-classical factors that are implicated in the pathogenesis of obesity, to clarify their potential role in the management of obesity and, where possible, to provide some practical clinical recommendations.

## 1. Introduction 

Obesity continues to be a major public health concern across the globe. According to the World Health Organization (WHO), overweight and obesity affect almost 60% of adults and nearly one in three children in the WHO European region [1]. Recent estimates suggest that overweight/obesity is the fourth most common risk factor for non-communicable diseases in the European region, after high blood pressure, dietary risks and tobacco [1]. Very alarming is also the fact that the prevalence of the disease increases through time with a dramatic rate. The prevalence of obesity in America rose from 6.8% in 1980 to 22.4% in 2019 and, similarly, in Europe, from 8.4% to 20% [2]. In addition, despite the potentially large benefits of weight loss, interventions have had small long-term success, especially at the population level, leading to a worldwide rise in overweight and obesity rates [3,4,5].

Although obesity is a multifactorial disease with genetic, environmental, behavioral and socioeconomic origins, a positive energy balance defined by excess energy intake and reduced physical activity has been traditionally viewed as a major classical factor promoting weight gain and the principal target of weight loss interventions. However, this association is not always straightforward, as it is further complicated by other parameters, such as diet quality and a sedentary lifestyle behavior [6]. Furthermore, these two parameters alone cannot justify the rise in the rates of obesity to epidemic levels which is observed in modern societies [7]. Further, an analysis of national data from the United States of America (USA) concluded that energy intake and physical activity of US adults have remained stable for almost 20 years, yet the levels of obesity have been constantly increasing [7]. This discrepancy suggests that other factors may be additionally implicated in body weight regulation, and that, despite not clearly and directly affecting the overall energy intake or expenditure, they cause a positive energy balance at the cellular adipocyte level [7]. A negative energy balance is considered a prerequisite for weight loss. Interestingly, some factors, such as diet quality and meal timing, can interfere with satiety signals and promote lean mass preservation and fat metabolism, therefore affecting the overall energy balance beyond strict caloric restriction [8]. As a result, a variety of dietary and non-dietary factors are implicated in the pathogenesis of obesity via complex mechanisms that involve distinct energy absorption, endocrine adaptations and interference with the circadian rhythms [7]. Some of them have been recognized as clearly increasing the risk of obesity, such as ultra-processed foods and endocrine-disrupting chemicals [9,10], while others may have protective actions against obesity, such as nuts, ingredients activating brown adipose tissue and the Mediterranean diet [11,12,13].

As previously stated, the classical approach against obesity involving caloric restriction and recommendations for increasing physical activity is not always successful and the disease’s prevalence continues to rise [4,5]. Therefore, there is this unmet need for effective health plans and strategies for the treatment of obesity. Understanding these non-classical aspects of obesity pathogenesis and attempting to incorporate them into our patients’ weight management programs may provide some additional benefit in the challenging fight against obesity. The objective of the present narrative review is to address factors implicated in the pathogenesis of obesity in a non-classical way. Certainly, some of these factors have been previously extensively studied and analyzed, while for others more evidence regarding their action is needed. However, our literature search did not retrieve a review focusing on an overall assessment of these factors. We believe that this review would be useful for clinicians of different specialties, as it will render them aware of certain issues that they may not be familiar with, as well as provide updated information regarding some already known and controversial topics. Furthermore, we provide concrete clinical recommendations and practical tips that could be incorporated in the weight loss programs of our patients based on the existing evidence.

## 2. Literature Search and Review Criteria

For the purposes of this narrative review, we conducted a search in PubMed electronic database for scientific literature published in English until February 2023. “Obesity pathogenesis”, “diet quality and obesity”, “dietary habits and obesity”, “non-dietary factors and obesity”, and “environmental factors and obesity” were used as search terms. Additional references were retrieved from reviewing the references cited in the original articles. Both human and animal studies were included in this review. The selection of factors was based on the available evidence and their practical implication in obesity treatment. For the presentation of the results, a categorization based on their relation to diet (diet related and non-dietary) was applied.

## 3. Diet-Related Factors

### 3.1. Diet Quality (What to Eat)

#### 3.1.1. Ultra-Processed Foods (UPFs)

According to the Food and Agriculture Organization, ultra-processed foods (UPFs) are formulations of ingredients, mostly of exclusive industrial use, that result from a series of industrial processes, such as drying, boiling, crushing, grinding, roasting, freezing, pasteurization, refrigeration, smoking, canning or non-alcoholic fermentation. These products are rich in fats, sugars, salts and cosmetic additives that render them unhealthy, yet convenient and highly palatable [14]. UPFs may account for more than half of the total energy intake in certain high-income countries, including USA and Canada [14], while recent data from Europe state that ultra-processed food and drink consumption rates range from 14% to 44% [15]. UPF intake has been positively associated with many non-communicable diseases including obesity, type 2 diabetes, dementia, cancer, and all-cause mortality [16,17,18,19].

The link between obesity and UPFs has been observed in observational studies [9] and prospective cohort studies [19]. Several pathophysiological responsible mechanisms have been proposed. Apart from their high caloric content and reduced potential for satiety that can lead to a positive energy balance, these products promote the development of obesity by disrupting delicate endocrine and metabolic pathways [9,19]. Some mechanisms that have been studied are endocrine adaptations regarding the circulating levels of insulin and leptin and their effect on energy storage, reduced postprandial thermogenesis and interaction with the gut–brain axis and the reward circuits that modulate energy intake [19,20]. Specifically, a clinical study has shown that when the participants consumed likeable foods rich in carbohydrate and fat, an increased striatal response was observed, compared to the consumption of equally caloric, likeable foods rich in fat or carbohydrates alone. This increased response could be associated with food craving and overeating of processed foods [21]. Another plausible explanation is that the structure and physical properties of UPFs are responsible for increased energy absorption from the colon. Interestingly, the importance of physical structure in energy absorption was evaluated in a clinical study where participants consumed peanuts in three different forms, whole peanuts, peanut butter or peanut oil, while the total amount of dietary fat remained stable. It was observed that the consumption of whole peanuts was associated with lower fat absorption compared to butter and oil (82% compared to 93% and 95%, respectively) [22]. Additionally, the included additives, as well as the packaging of UPFs, alter gut microbiota and contribute to dysbiosis, a state that has been associated with obesity through various mechanisms [9,19]. The importance of these non-nutritional components of a diet rich in UPFs has been demonstrated in a prospective cohort of 22,895 participants. In this cohort, where two distinct food classification systems were evaluated, higher mortality risk was related to food processing, but not to poor nutritional quality [23]. In conclusion, it is clear that UPFs can promote the development of obesity through a variety of mechanisms and dietary guidelines discourage their consumption [24]. It is therefore important that clinicians educate their patients in recognizing which foods are UPFs and, also, in understanding their harms and limiting their consumption.

#### 3.1.2. Mediterranean Diet

The Mediterranean diet (MedDiet) is a dietary pattern that originates from the countries of Southern Europe. This pattern is defined by a high intake of vegetables, fruits, nuts, and whole grain cereals, the use of olive oil as the primary source of fat, a moderate intake of wine, especially with meals, and a low intake of red meat and saturated fats [25,26]. Numerous studies have been published emphasizing the importance of MedDiet in the prevention and management of chronic diseases such as cardiovascular diseases, cancer, depression, neurodegenerative diseases, obesity-related disorders such as diabetes mellitus and non-alcoholic fat liver disease and, of course, obesity per se [26,27,28,29,30]. It is believed that the beneficial effects of MedDiet arise from the synergistic interactions between its various components (including fats, starch, fibers, vitamins and bioactive molecules such as phytosterols, polyphenols and terpenes) and are extended beyond the actions of single compounds [27]. Interestingly, the relatively high content of fat in MedDiet has been considered by some investigators as a concern, as it has been speculated that it could lead to weight gain; however, this hypothesis has not been validated. On the contrary, accumulating evidence from systematic reviews and meta-analyses underscore that adherence to MedDiet is associated with modest weight loss and improvements in markers of central adiposity, such as waist circumference [31,32,33,34]. In a meta-analysis of 19 randomized controlled trials (RCTs), adherence to MedDiet significantly reduced body weight; this effect was stronger when MedDiet was followed for more than 6 months and also, when combined with energy restriction and increased physical activity [32]. Thus, taking into consideration the existing evidence, it seems that MedDiet is a healthy dietary pattern with several benefits in weight management and its adoption should be encouraged.

#### 3.1.3. Nuts Intake

Nut consumption is generally considered as a “healthy habit” and nuts are essential components of certain dietary patterns that confer substantial health benefits, such as the Mediterranean diet and the Dietary Approaches to Stop Hypertension (DASH) diet [26,35]. Nuts are rich in dietary fiber, monounsaturated fatty acids (MUFAs), vitamins and bioactive compounds such as phytosterols and are favorably associated with several health outcomes, including coronary heart disease and diabetes [36]. However, nuts are energy-dense foods, and concerns have been raised about their potential obesogenic effect. Therefore, their consumption is advised in limited amounts. Contrary to this common notion, evidence from RCTs and meta-analyses does not support an obesogenic action of nuts; it is rather proposed that nut consumption may be protective against obesity. A network meta-analysis of 105 RCTs aimed to analyze the effect of tree nuts and peanuts on markers of adiposity, such as waist circumference, body weight, body mass index (BMI) and body fat percentage. Nut consumption was not associated with an increase in the aforementioned parameters (except for an increase in waist circumference with diets high in hazelnut); on the other hand, almond consumption was related to a reduction in waist circumference [37]. This result is consistent with earlier research demonstrating the positive benefits of nut consumption on long-term weight change and blood lipids [38,39]. Furthermore, a meta-analysis and meta-regression of prospective studies and RCTs reported an inverse relationship between nut consumption and body weight [11]. A possible explanation of this inverse association is that nuts often replace other snacks that are high in sugars and fats [38]. Moreover, additional mechanisms have been postulated. Despite being energy-dense, nuts are less bioaccessible due to their physical properties and their fiber content, leading to less energy absorption [11]. Additionally, the consumption of nuts requires effort for oral processing, which, along with their high fiber content, delays gastric emptying and promotes satiety. Finally, nuts are rich in MUFAs and polyunsaturated fatty acids (PUFAs), which are believed to increase postprandial thermogenesis and energy expenditure [11]. Thus, according to the existing evidence, the daily consumption of nuts as part of a healthy diet should not be discouraged in the fear of promoting weight gain. On the contrary, provided they are consumed in moderation, these natural products could be valuable assets in the fight against obesity.

#### 3.1.4. Unrefined Compared to Refined Grains

An increased whole grain consumption has been consistently promoted as a key component of a healthy dietary pattern in recent decades, as it has been linked to a reduction in non-communicable diseases and all-cause mortality [40]. Specifically, the Dietary Guidelines for Americans recommend that at least half of the grains that are daily consumed should be whole grain [41]. The association between whole grain consumption and obesity has been extensively studied in observational studies and RCTS and the results are often conflicting. Consumption of high-fiber whole grain foods was associated with less weight gain over a 12-year follow-up period in a prospective cohort of 74,091 female nurses [42]. Furthermore, in a prospective cohort of middle-aged adults, eating at least three servings of whole grains per day resulted in a 10% lower volume of visceral adipose tissue, a marker of central obesity [43]. Apart from observational studies, the relation between whole grains and several metabolic parameters has been evaluated in RCTs. In a RCT of 81 participants, whole grains increased resting metabolic rate and stool energy excretion, effects that translated to a 92 kcal higher net daily energy loss [44], while in a small RCT of 14 subjects, a diet with approximately 5–6 servings of whole grain per day increased whole body protein turnover and net protein balance [45]. Additionally, a RCT that evaluated the metagenomics profile of gut microbiota after 8 weeks of dietary intervention rich in whole grains concluded that, despite no significant changes in the microbiome, weight loss and attenuation of systemic low-grade inflammation were observed [46]. Several mechanisms concerning the effect of whole grain intake on energy regulation have been proposed, including the reduction in energy intake (due to attenuation of energy absorption from the gastrointestinal track and increased satiety), the action of certain ingredients of whole grains (such as minerals and phytosterols) on adipocyte functions and thermogenesis, as well as the fermentation by the gut microbiota and the production of secondary metabolites, such as short-chain fatty acids [47,48]. Despite the positive results from observational studies and the plausibility of the aforementioned mechanisms, evidence from systematic reviews and RCTs is rather inconclusive and causality cannot be proven [49,50]. Nonetheless, in accordance with current dietary guidelines, health professionals should promote the consumption of whole grains and should assist their patients in incorporating them in their daily routine (by recommending, for instance, the substitution of white bread, plain flour and regular pasta by their whole grain versions).

#### 3.1.5. Macronutrients: Low-Carbohydrate and Low-Fat Diets

The importance of distinct macronutrients (carbohydrates, fat and protein) in human metabolism and weight management has been addressed in several studies. The scientific hypothesis is that isocaloric diets with different proportions of macronutrients, such as low-carbohydrate or low-fat diets, will lead to distinct energy balances [51]. There are several underlying pathophysiological mechanisms in support of this hypothesis, including discrepancies in energy absorption, effects on gut microbiota, postprandial thermogenesis and secretion of hormones and peptides regulating metabolism of nutrients and satiety [8,51,52,53,54,55]. Despite being intriguing, this hypothesis cannot be robustly supported by the existing evidence, as studies that compare low-fat with low-carbohydrate diets often yield conflicting results. A meta-analysis of 38 RCTs and 6449 participants with BMI ranging from 22.0 to 43.9 kg/m^2^, concluded that low-carbohydrate diets were associated with a more significant weight reduction at 12 months; however, this effect was attenuated at 24 months [54]. Similarly, a more recent meta-analysis of 3939 overweight and obese participants, that included 33 RCTS with duration of at least 6 months, reported that low-carbohydrate diets were more effective at weight loss compared to low-fat diets; nevertheless, no difference was observed after 24 months [53]. On the contrary, it seems that the most important attribute of a diet is whether it consists of healthy foods, such as whole grains and unsaturated fats, instead of unhealthy foods, such as refined sugars and saturated fatty acids (SAFAs). A prospective cohort study of 37,233 adults showed that both low-fat and low-carbohydrate diets were associated with increased mortality when they comprised of unhealthy foods, while the opposite effect was observed for both diets when they were based on healthy foods [56]. In addition, it should be noted that in the case of very low-carbohydrate diets, the fat content is necessarily very high. The long-term effects on health and the cardiovascular system, especially when the included fats are mostly of animal origin, represent a serious concern [57]. In conclusion, current evidence tends to downgrade the significance of the macronutrient content of the diet and supports that macronutrient composition is not important for long-term weight loss. Clinicians should emphasize to their patients that in order to achieve long-term, sustainable weight loss, they should focus on the overall quality and caloric content of their diet instead of the distinct macronutrients.

#### 3.1.6. Nutritional Ingredients Associated with White Adipose Tissue (WAT) Browning and Brown Adipose Tissue (BAT) Activation

In humans, three types of adipose tissue have been identified: white, brown, and brown-like or beige adipose tissue. White adipose tissue (WAT) is the most abundant type, and its primary functions are energy storage and adipokine secretion. Brown adipose tissue has been found in adult humans in small amounts (mostly in the cervical-supraclavicular region) and has thermogenic properties, which are mediated by the expression of the uncoupling protein 1 (UCP1). Beige adipocytes express UCP1 and induce thermogenesis, but have different origins from the brown ones; specifically, they develop in white adipose tissue depots under certain stimuli, such as cold exposure and adrenergic stimulation, a process known as “browning” [12,58]. The induction of thermogenesis and the subsequent increased energy expenditure has been considered as a potential strategy in the management of obesity [59]. Interestingly, several bioactive nutritional elements have been studied as possible modulators of BAT activation and WAT “browning”. Some of these compounds are capsaicin and capsinoids, resveratrol, green tea catechins, berberine, curcumin, omega-3 PUFAs, menthol and retinoic-acid. These molecules have been tested in experimental settings and found to induce thermogenesis by modulating multiple signaling pathways [12,58,59,60,61]. However, for a variety of reasons, translating these findings into clinical practice remains very difficult. First and foremost, high-quality human studies are lacking, as the majority of evidence comes from cell lines and experimental trials. Furthermore, these molecules have been tested at extremely high doses, making it unclear whether such doses could be used and whether bioavailability or safety issues would arise. Finally, the true benefit of increased thermogenesis in humans, as well as the potential activation of counter-regulatory mechanisms such as increased energy intake, must be determined [59]. In conclusion, despite the intriguing concept and pathophysiological mechanisms involved, the use of molecules aimed at BAT activation or WAT “browning” for the management of obesity cannot be recommended in clinical practice until the aforementioned issues are resolved.

### 3.2. Dietary Habits (When to Eat)

#### 3.2.1. Intermittent Fasting

In recent years, intermittent fasting diets have gained popularity as weight loss strategies, as they are typically simpler and easier to adhere to than conventional calorie-restricted diets [62]. There are various diverse intermittent fasting protocols. Alternate-day fasting (ADF), 5:2 fasting, and time-restricted eating (TRE) have been examined the most [62,63]. In the zero-calorie ADF protocol, a day of total fasting is followed by a day of unlimited eating, while in the modified ADF protocol, a day of decreased caloric intake (no more than 500–600 kcal) is followed by a day of unrestricted eating. The 5:2 pattern consists of 2 consecutive or non-consecutive days in which calorie intake is restricted to 500–1000 kcal, followed by 5 days of unrestricted eating (feast days). TRE does not restrict caloric intake; rather, eating is restricted to specific times of the day. The most common TRE schedule consists of 16 h of fasting and 8 h of eating (16:8). Early TRE is a variation of TRE in which calorie intake is restricted to the morning and lunch time and no meals are consumed after 3:00 p.m. [62].

The metabolic switch from glucose to ketones is considered a key mediator of the health benefits of intermittent fasting. Hepatic glycogen depletion after prolonged fasting triggers utilization of fat as energy fuel, oxidation of free fatty acids and production of ketones [62,64,65]. Numerous advantageous metabolic adaptations have been attributed to intermittent fasting including enhanced mitochondrial function, attenuation of oxidative stress, downregulation of anabolic processes such as lipogenesis, increased hepatic and skeletal muscle glycogenolysis and modulation of adipokine secretion (elevated adiponectin and lower levels of leptin) [63]. Chrononutrition and synchronization of food intake with the biological circadian rhythms are also studied as mediators of the health benefits of TRE, particularly when eating windows are restricted to daylight hours [62]. In addition, an unvoluntary restriction of caloric intake in feeding periods has been documented [65]. Changes in gut microbiota that provoke white adipose tissue browning and increased energy expenditure via non-shivering thermogenesis have also been reported in an experimental study of intermittent fasting [66].

Several studies and meta-analyses on the effect of intermittent fasting in various populations have been published. Their thorough analysis is beyond the scope of this review. In general, current evidence suggests that intermittent fasting may be an effective weight management approach and may confer certain cardiometabolic benefits, including improvements in insulin resistance, lipid profile and oxidative stress [62]. Indicatively, an umbrella review of 11 meta-analyses and 130 RCTs concluded that intermittent fasting, especially ADF, may be employed as a weight loss approach for adults with overweight and obesity [67]. A recent meta-analysis of 11 RCTs that evaluated intermittent fasting (excluding TRE regimens) against continuous energy restriction revealed that intermittent fasting was superior to continuous energy restriction in terms of weight loss, but there was no significant difference in BMI. However, there are limitations in this research, including the heterogeneity of the included studies due to small sample sizes and varying follow-up lengths [68]. In a recent study conducted over 6 months in 547 participants, it was shown that the number of meals per day was positively associated with weight change. The authors concluded that their findings do not support the use of time-restricted eating as a strategy for long-term weight loss in a general medical population [69]. Another study, however, in favor of TRE, showed that late isocaloric eating has been associated with increased hunger, altered gene expression that promotes lipid storage, increased leptin levels and reduced energy expenditure [70]. In contrast, a short-term trial concluded that both the morning and evening-loaded isocaloric diets led to identical weight reduction, but the morning-loaded diet was associated with less subjective hunger and appetite, suggesting possible behavioral changes [71]. Additionally, safety concerns surrounding the probable relationship between intermittent fasting and specific outcomes such as infertility and eating disorders remain unresolved [62]. In addition, there are unresolved clinical questions, the most intriguing of which is whether intermittent fasting regimens are superior to traditional continuous energy restriction. Existing research does not show any superiority of intermittent fasting at this time [62]. Thus, intermittent fasting appears to be a promising strategy against obesity that could be beneficial for certain populations, while high-quality long-term research is still required. Such regimens may be offered to patients who find them easier to follow, but they should not be proposed as a superior alternative to continuous energy restriction.

#### 3.2.2. Skipping Breakfast

Many authors consider that breakfast is the most important meal of the day [72]. Some studies have associated skipping breakfast with several health outcomes, including the development of obesity, dyslipidemia, type 2 diabetes, cardiovascular and all-cause mortality [73]. In a meta-analysis of 45 cross-sectional and cohort studies, breakfast skipping increased the risk of overweight and obesity, as well as abdominal obesity [74]. In addition, in a cross-sectional analysis of 23,758 participants, eating breakfast was negatively associated with obesity and Dietary Inflammatory Index (DII), a score developed to quantify the inflammatory burden of a diet [75]. However, when the importance of breakfast consumption in weight management is evaluated in RCTs, the results are often contradictory. A meta-analysis of RCTs found that breakfast skipping may lead to a significant but small weight loss of 0.5 kg but no significant differences in other cardiometabolic parameters, except for a rise in low-density lipoprotein (LDL) cholesterol [76]. Another meta-analysis of 13 RCTs concluded that breakfast skippers had a lower total energy intake and a small difference in weight compared to participants who consumed breakfast. The authors acknowledge that there was inconsistency across the trials results and that their findings should be cautiously interpreted; nevertheless, they suggest that the inclusion of breakfast might not be a good weight loss strategy [77].

Several mechanisms linking breakfast skipping and obesity have been postulated, including decreased satiety, which could lead to overeating, impaired insulin sensitivity after meals throughout the day [78], as well as differences in postprandial thermogenesis [79]. Behavioral factors could also be implicated, as breakfast consumption may indicate a healthier and more active lifestyle [80]. Moreover, the quality of breakfast seems to play an important role, as the consumption of a “healthy breakfast”, consisting of fruits and fiber-rich carbohydrates may be the cause of the aforementioned beneficial metabolic effects [78]. In a Swiss cross-sectional study, a “prudent” breakfast consisting of fruits, unprocessed and unsweetened cereal flakes, nuts/seeds and yogurt was negatively associated with abdominal obesity. This association could be due to the adoption of a healthy dietary pattern throughout the rest of the day [81]. In conclusion, the evidence regarding the clinical importance of breakfast in the management of obesity is conflicting and more well-designed studies focusing on the composition of breakfast and its long-term effects are required. Until then, clinicians should focus on the quality of foods that are consumed as breakfast and educate their patients in adopting a “healthy” breakfast that contains for example, fruits, yogurt and whole grains instead of an “unhealthy” breakfast rich in refined sugars and trans fats.

### 3.3. Speed of Eating (How to Eat)

“Eating slowly” has been traditionally advocated as a healthy eating behavior that would protect individuals against obesity. Interestingly, this argument is not just an anecdotal advice; instead, scientific evidence points to that direction too. The association between the speed of eating and the risk of obesity was investigated in a meta-analysis of 23 epidemiological studies [82]. The included studies were cross-sectional and longitudinal and the rate of eating was evaluated with self-reporting measures in 22 studies and with an eating monitor in 1 study. This meta-analysis reported that increased speed of eating was associated with significantly higher BMI and increased risk of being obese [82]. In addition, a more recent narrative review suggested that faster eating could increase the risk of abdominal obesity and metabolic syndrome in children and adults [83]. A plausible underlying pathophysiological mechanism is that faster eating could lead to excess energy intake due to reduced oral processing and delayed transfer of satiety signals from the gastrointestinal tract to the brain (gut–brain axis) [82,84,85]. Distinct eating behaviors provoke changes in the secretory patterns of the enteroendocrine hormones, such as glucagon-like peptide-1 (GLP-1), peptide tyrosine tyrosine (PYY) and ghrelin. Further, slower eating has been associated with higher secretion of the anorexic hormones PPY and GLP-1 [84], as well as lower levels of ghrelin in some studies [85]. This link between eating rate and energy intake was studied in a meta-analysis of controlled laboratory trials; this meta-analysis reported that slower eating was associated with less energy intake compared to faster eating [86]. A clinical question that arises after the consideration of the above data is whether the manipulation of eating behavior through interventions and educational programs could be feasible and efficient against obesity. A narrative review that addressed this matter of eating manipulation concluded that, in most cases, the energy intake and the feeling of fullness is not modified in subsequent meals, neither is the intermeal interval; therefore, it is still unclear whether acute changes that occur after eating manipulation would provoke significant long sustained changes in weight [87]. In addition, a very recent study found that a 5-week intervention of lessons that aimed to modify eating behavior of overweight and obese women led to reduced laboratory measured eating rate and prolonged meal duration; no changes in overall energy intake or body weight were observed. As the authors highlight, it is possible that a multifactorial approach that would combine eating pace instruction with diet and physical activity could be useful [88]. Thus, it can be said that current evidence supports that increased speed of eating is associated with certain pathophysiological changes that could promote obesity. Further research from long-term and high-quality studies is needed for these findings to be incorporated in clinical practice interventions and guidelines. Until then and due to the plausibility of the pathophysiological mechanisms, “eat slowly” may be prudent advice for patients in everyday clinical practice.

## 4. Non-Dietary Factors

### 4.1. Endocrine-Disrupting Chemicals and Microplastics

Endocrine-disrupting chemicals (EDCs) have been defined as “exogenous chemicals, or mixture of chemicals, that interfere with any aspect of hormone action” [89]. The hypothesis that EDCs could be associated with obesity originated in 2002 and gave rise to the term “obesogens”, which are chemicals that lead to the accumulation of white adipose tissue, in vivo, after exposure [90]. These compounds are present in many products, including pesticides, cosmetics, toys, clothes, pharmaceuticals and foods [10,90,91]. A wide range of chemicals have been identified as obesogens, some of which are bisphenol A, carboxylmethylcellulose, dibutyltin and acrylamide, and the list is being updated [92]. Furthermore, the high concentration of microplastics in the environment and human exposure to them has also been associated with toxic effects and obesity, raising another serious matter of public health [93]. An important mechanism of action of obesogens is the activation of peroxisome proliferator-activated receptor γ (PPARγ) and its heterodimeric partner, the 9-cis retinoid X receptor (RXR). Moreover, other actions have been suggested, such as epigenetic modifications, changes to chromatin architecture, gut microbiome modifications, as well as interference with various signaling pathways and hormones [90]. As a result, there is an increased commitment of adipocytes, which, apart from being abundant, are also dysfunctional, contributing to insulin resistance and inflammation. Furthermore, a metabolic switch towards energy storage, gut dysbiosis and transgenerational effects that promote obesity have also been postulated [90].

Due to the serious health effects of EDCs on metabolism and public health in general, regulatory authorities have been established to minimize the associated risks. The regulation on the registration, evaluation, authorization and restriction of chemicals (REACH) is a European Union regulation that addresses the manufacture and use of chemical substances, as well as their potential effects on human health and the environment [94]. The Endocrine Society, however, expresses its concerns regarding REACH and underlines the need for drastic measures. These measures include enacting regulatory decisions that protect public health and are constantly updated in response to new scientific data, paying special attention to vulnerable populations such as pregnant women and infants, understanding and implementing in research and regulation that the deleterious effects of EDCs can be observed even in low-dose exposure and avoiding equally dangerous substitutions [95]. A petition endorsed by 44 organizations, representing the European and international endocrine research and clinical community, was recently published highlighting the need for an urgent revision of REACH by June 2023 [96].

### 4.2. Psychotropic Medications

The prevalence of overweight and obesity is much higher in people with serious mental disorders compared to general population, reaching up to 60% [97]. Psychotropic medications play an important role in the etiology of obesity in this subpopulation, primarily by increasing appetite and food intake and delaying satiety. These actions are largely due to antagonistic activity at the serotoninergic 5-HT2C and histaminergic H1 receptors [98]. Weight gain varies by psychotropic medication subcategory, with second generation antipsychotics, such as olanzapine and clozapine, having the strongest effect and increasing body weight beyond the threshold of 7%, which is defined as clinically significant by the Food and Drug Administration (FDA) [98,99]. On the other hand, antidepressants have milder effects compared to antipsychotics and lead to weight gain ≥ 5% [98,99]. The management of weight gain in patients on psychotropic medication is important, because in addition to the negative effects on metabolic health and all-cause mortality, a decrease through time in treatment response has been observed [98]. Despite the possibility of low adherence due to reduced motivation, lifestyle intervention measures such as physical activity and nutrition counseling should be offered to these patients [100]. The use of agents that will prevent weight gain such as metformin, GLP-1 analogues or topiramate is also proposed, but the supporting evidence is of low quality [98,101,102]. Another strategy is switching antipsychotic medications. This strategy was evaluated in a recent systematic review and meta-analysis of 61 studies and 8554 participants and the authors concluded that switching to agents with more favorable profile, such as aripiprazole and ziprasidone, was associated with improvement in weight and cardiometabolic outcomes; however, the potential risk of patient destabilization and relapse should be balanced against the aforementioned benefits [103]. Thus, it is clear that psychotropic medications significantly contribute to the burden of obesity in modern societies and appropriate measures should be implemented. Clinical vigilance and close collaboration between the treating psychiatrists and the weight management professionals are crucial.

### 4.3. Sleep Quantity and Quality

Sleep restriction is common in modern societies and, according to Center for Disease Control and Prevention (CDC), one-third of adults in USA sleep less than recommended [104]. The parallel increased prevalence of sleep restriction and obesity raises the question of whether these two entities have a causal relationship [105]. Evidence from cohort studies indicates that lower sleep duration is related to a higher risk of obesity [106]. Furthermore, a systematic review and meta-analysis of prospective cohort studies that explored the relationship between short sleep and a variety of health outcomes found a correlation between sleep restriction and obesity [107]. Several underlying mechanisms have been proposed. Specifically, sleep restriction in humans results to an increased energy intake, while having a relatively minor effect on energy expenditure [108,109]. This impact may be mediated through hormonal changes that stimulate hunger, including an increase in the ratio of ghrelin to leptin and of endocannabinoids, as well as a decrease in GLP-1 [106,108,110]. In addition to hormone-related alterations that encourage hunger-eating, hedonic-eating also appears to be upregulated by the activation of certain neural networks that make a person susceptible to making unhealthy food choices [105,108]. At this point, it is important to note that not only the quantity of sleep in means of duration, but also the quality of sleep is a variable that could be associated with increased body weight [111]. Sleep fragmentation is a sleep condition that could be caused by environmental factors, such as caffeine and alcohol consumption, and by sleep disorders such as apneas. When objectively measured with in-home polysomnography, sleep fragmentation was positively associated with obesity in a multicenter cohort study of 5723 participants [112].

Most evidence regarding sleep deprivation and obesity arises form observational studies and experimental data, and results from RCTs are often conflicting. A meta-analysis of RCTs reported a significant increase in subjective hunger, energy intake and weight gain after sleep restriction, which was not accompanied by a significant change in the appetite-regulating hormones [105]. After taking into consideration the aforementioned data, a plausible clinical question that arises is whether sleep extension could have favorable effects in metabolism and markers of obesity. This question was recently investigated in a RCT of 80 overweight participants that regularly slept less than 6.5 h. The lengthening of sleeping hours following sleep hygiene counseling sessions was related with a calorie intake that was approximately 250 kcal less than that of the control group. There were no substantial variations in energy expenditure, and a net negative energy balance was established [113]. This well-designed RCT reinforces the findings from previous prospective and intervention studies regarding the positive impact of sleep extension on weight status [106,108]. In conclusion, existing evidence suggests that sleep deprivation is a potential contribution to the obesity epidemic in modern societies. However, more robust evidence from randomized controlled trials is still required to elucidate the implicated mechanisms and examine the potential benefits of sleep management tactics. Nonetheless, health professionals should emphasize to their patients that adequate sleep (at least 7 h of sleep for adults aged 18–60) is an essential part of a healthy lifestyle [114]. In cases of sleep disturbances, collaboration with other specialties and incorporation of sleep hygiene strategies into the weight loss program may be helpful for overweight and obese people.

### 4.4. Light at Night

Exposure to light at night (LAN) is another factor that could be implicated in the pandemic rise of obesity in modern societies. Evidence in support of this hypothesis initially arose from experimental studies and was further enriched by studies in shift-workers and general population too [115,116,117]. In more detail, a population-level study that analyzed exposure to artificial LAN (as assessed by satellite images) in relation to the prevalence rates of overweight and obesity in more than 80 countries, found a significant positive association between these two parameters [118]. Similar findings were reported in a more recent, large prospective study, where LAN exposure estimated also by satellite images increased the risk of developing obesity over a 10-year period in middle-aged to older USA adults [116], as well as in a prospective analysis of 43,722 women [119]. Moreover, LAN has been found to increase the risk of metabolic syndrome [120], as well as diabetes and hypertension [121]. Interestingly, a cross-sectional study from China which included 98,658 participants stated that LAN exposure was negatively associated with glucose homeostasis, even after adjusting for several risk factors including BMI [122]. Several possible pathophysiological mechanisms that could support the relation between LAN and obesity have been proposed. Chronic exposure to LAN interacts with the suprachiasmatic nucleus of the hypothalamus, which is the master circadian regulator, and provokes altered expression of clock genes, disruption of circadian rhythms and subsequently, metabolic abnormalities [115,123]. Furthermore, LAN suppresses melatonin expression from the pineal gland. Melatonin has been characterized as the “hormone of darkness”, as it is normally expressed at night and suppressed at daytime. This hormone is implicated in the synchronization of the circadian rhythms and is also believed to have beneficial metabolic actions; low levels of melatonin have been associated with obesity, as well as with glucose dysregulation [115,121]. In addition, apart from these actions in the brain, acute LAN exposure has been found to affect peripheral tissues, including liver, pancreas, adipose tissue and adrenal cortex [123]. In conclusion, exposure to LAN has rapidly increased in recent years due to external light pollution and the use of electronic devices such as televisions and smart phones at home; therefore, clinicians must inform and educate their patients about the potentially associated metabolic harms.

## 5. Limitations, Knowledge Gaps and Area for Future Research

This review aims to provide an overview of dietary and non-dietary factors that are implicated in the pathogenesis of obesity in a non-classical way. We believe that this approach could be useful in the treatment of obesity, especially when taking into consideration that the classical approach (guidance for reduced caloric intake and increased physical activity) often fails to provide satisfactory results. However, this review has some limitations that need to be acknowledged. First of all, this is a narrative, non-systematic review. Furthermore, our literature search was based on “phrases” (i.e., obesity pathogenesis, diet quality, dietary habits, etc.) and not on text words. This method retrieves fewer results; therefore, some eligible studies might have been missed. We tried to conduct a comprehensive search of the literature and to include several factors that are related to this review’s subject. Nonetheless, some additional factors that could also be related to obesity in a non-classical way may have been omitted. Moreover, as already mentioned, despite the plausibility of the pathophysiological mechanisms, the quality of the supporting evidence of some analyzed factors is low, meaning, therefore, the absence of concrete recommendations. For example, UPFs and EDCs have been recognized as contributors to the obesity pandemic and their limitation is supported by robust evidence [24,95]. On the other hand, other factors, such as light at night, breakfast and speed of eating, are more “ambiguous” regarding their actual impact on weight management. Nevertheless, there is no doubt that the unmet need for effective measures against obesity is present and future research derived from well-designed studies that will clarify these aspects is needed.

## 6. Conclusions

Obesity is a chronic, multifactorial disease which imposes a substantial burden on patients and health care systems. The epidemic rise of obesity in modern societies is a serious health matter and the classical approach that focuses solely on decreasing the ingested calories and increasing physical activity often yields suboptimal results. The existence of some dietary and non-dietary factors that could be involved in the pathogenesis of obesity in a non-classical way, as well as the associated limitations and evidence gaps, have been analyzed.

Conclusively, a simplified approach for clinicians in the field of weight management that would take into consideration these non-classical aspects of obesity pathogenesis could be proposed. First of all, the importance of a negative energy balance in weight loss should always be emphasized and prioritized. On top of that, matters of diet quality and dietary behavior could be discussed with our patients, starting from practices that have been incorporated in official recommendations [41,124,125,126] as beneficial for weight loss and nutrition in general. These include the avoidance of UPFs, the incorporation of unrefined grains and nuts in the diet and the adoption of a healthy dietary pattern such as the Mediterranean diet. Furthermore, despite the lack of concrete evidence and specific recommendations, the advice of “slow eating” is prudent. The significance of the macronutrient content of the diet should be downgraded. Instead, the superiority of the overall caloric content and quality of the diet against the ratios of distinct macronutrients should be underlined. Similarly, when asked about the necessity of breakfast, clinicians should emphasize the importance of a healthy breakfast instead of a “western” breakfast. Additionally, the option of intermittent fasting or time-restricted eating should be offered to certain patients who are eager to follow it. Non-dietary factors should be addressed too. A prudent approach would be to inform patients about the importance of sleep and to advise them to sleep at least 7 h per day, according to existing guidelines. Likewise, a recommendation to limit exposure to light at night could be sensible, despite the lack of robust evidence, based only on the pathophysiological mechanisms and the simplicity of this advice (for example turning off smart phones and television during sleeping hours, avoiding street light if possible). The serious matter of EDCs is maybe more difficult to address at a personal level and depends mostly on the establishment of legislation and the implementation of public health strategies such as REACH. Nevertheless, patient education and vigilance, regarding, for instance, the products that usually contain EDCs and how they should be avoided is also important. In conclusion, the multifactorial nature of obesity, its rising prevalence and its negative consequences cannot be denied. Taking these facts into consideration, we believe that attempting to address these non-classical aspects of the obesity pathogenesis could provide some additional benefits for both patients and health care systems in terms of obesity treatment.

## Data Availability

No new data were created or analyzed in this study. Data sharing is not applicable to this article.
